# Incidence and treatment of intracapsular femoral neck fractures in Germany

**DOI:** 10.1007/s00402-022-04504-3

**Published:** 2022-06-23

**Authors:** Dominik Szymski, Nike Walter, Siegmund Lang, Susanne Baertl, Johannes Weber, Volker Alt, Markus Rupp

**Affiliations:** Department of Trauma Surgery, University Medical Centre Regensburg, Franz‑Josef‑Strauss Allee 11, 93053 Regensburg, Germany

**Keywords:** Hip fracture, Intracapsular femoral neck fracture, Incidence, Femur, Operative treatment

## Abstract

**Introduction:**

Intracapsular femoral neck fractures are one of the most common fractures in Germany. Nevertheless, the epidemiology and treatment modalities are not described comprehensively. For this reason, this study highlights the epidemiology of femoral neck fractures in different age groups and summarizes treatment strategies within the period from 2009 to 2019 based on nationwide data.

**Materials and methods:**

In this retrospective cohort study all cases of intracapsular femoral neck fractures (ICD-10: S72.0) between 2009 and 2019 in Germany were analyzed with regard to epidemiology, incidence and treatment. Operation and procedure classification system (OPS)- codes in combination with intracapsular femoral neck fracture as main diagnosis were taken to investigation. Data was provided by the Federal Statistical Office of Germany (Destatis).

**Results:**

A total of 807,834 intracapsular femoral neck fractures with a mean incidence of 110.0 per 100,000 inhabitants annually was detected within eleven years. In 68.8% of all fractures patients were female. Most patients were older than 70 years (82.4%), and 56.7% were older than 80 years. The overall increase of fracture numbers between 2009 and 2019 was 23.2%. Joint replacement has been most often performed (80.4%). Hemiarthroplasty (56.8%) and total hip arthroplasty (22.8%) were the most common procedures with an increase of 27.1 and 38.6%, respectively. The proportion of cemented hemiarthroplasties was 86.2% while 51.3% of all total hip arthroplasties were totally or partially cemented. Osteosyntheses were mainly conducted using dynamic compression screws (34.0%), conventional screws (31.3%) and nails (22.2%).

**Conclusion:**

The incidence of intracapsular femoral neck fractures in Germany has been increasing continuously within the last decade. In particular, patients over 80 years suffered predominantly from this type of fracture. The majority was treated with a joint replacement procedure, mainly cemented hemiarthroplasty.

## Introduction

Intracapsular femoral neck fractures are among the most common fractures representing around 12% of all fractures in Germany with increasing incidence in the last decade [1]. Already in 1997, Gullberg et al. calculated that the number of hip fractures worldwide would double from 1990 to 2025 and double again by 2050 with a range between 7.3 and 21.3 million fractures worldwide [2]. Mostly femoral neck fractures affect the elderly population and are the result of a low-energy trauma. A more uncommon mechanism of injury are high-energy trauma situations such as motor vehicle accidents. Especially younger patients suffering from femoral neck fractures have experienced such a high-energy trauma [3]. General risk factors for femoral neck fractures are female sex, white race, increased age, low estrogen levels, tobacco or alcohol abuse and falling tendency [3–5].

The high mortality rate demonstrates the severity and burden of this injury for the population. Major and North reported an in-hospital mortality of 7.5% for femoral neck fractures with a proportion of 2.1% preoperatively and 5.4% postoperatively in 2016 [6]. Within the first 90 days after injury a 9.6% mortality rate was determined with an significant association regarding increased age, male sex and increased time between accident and surgery [7].

An operative treatment of femoral neck fractures is mostly obligatory, because of instable fracture situations and a decrease of complications by fast mobilization. Basically, a decision has to be made between osteosynthesis and (partial) joint replacement. The main objective of an osteosynthesis is the repositioning in anatomical position followed by stable fixation of the fracture. Different methods are available to achieve this purpose. Usually dynamic compression screws (sliding hip screw–SHS), dynamic hip screws with a blade or antirotation screw or multiple cannulated screws are used as osteosynthetic devices [3, 8]. For elderly patients with displaced femoral neck fracture and preexisting signs of osteoarthritis an arthroplasty is the gold standard in fracture treatment. Depending on the general condition and physical demands, a hemiarthroplasty or total hip arthroplasty is performed [9].

While for some countries a reduction in the incidence of femoral neck fractures was described [10, 11], other nations reported an increase of the incidence [12–14]. A growth of the incidence rate was especially documented in high-income countries as a result of a steadily ageing population [5, 13–15].

Therefore, aims of this study were (1) to provide detailed information about the epidemiology and incidence of femoral neck fractures in Germany within the last decade from 2009 to 2019. (2) Further age and sex dependent incidence differences should be analyzed. (3) Lastly, current treatment practice depending on age and sex should be elucidated comparing data from 2019.

## Materials and methods

In this retrospective cohort study, cases of femoral neck fractures and the surgical treatment were analyzed based on data from all German medical institutions between the years 2009 and 2019 provided by the Federal Statistical Office of Germany (Destatis). Patient data of the ICD-10 codes ‘S72.0: femoral neck fracture’ were used to identify hospitalized patients aged 20 years or older diagnosed with a femoral neck fracture within the 11-year time period. Thereby, a detailed analysis of epidemiology with focus on age groups and sex was obtained. The age groups were divided into 10-year increments. For all cases with a main diagnosis of a femoral neck fracture (ICD-10: S72.0) the surgical treatment coding was used (OPS-Codes = operation and procedure codes) to report the type of osteosynthesis or arthroplasty (Table 1). The fixation method was also analyzed using the OPS code evaluation. Due to a missing OPS code for conservative therapy, data on treatment of femoral neck fractures could be only analyzed for surgical procedures.

**Table 1:** Used OPS-Codes for the analysis of surgical treatment for intracapsular femoral neck fractures (ICD-10: S72.0) and classification of procedure

Procedure	Used OPS-Codes
Osteosynthesis through screw	5–790.0e
Osteosynthesis through dynamic compression screw	5–790.8e
Osteosynthesis through wire	5–790.1e 5–790.2e
Osteosynthesis through nail	5–790.3e 5–790.4e 5–790.5e 5–790.ce
Osteosynthesis through fixator	5–790.6e 5–790.de 5–790.me 5–790.pe
Osteosynthesis through plate	5–790.7e 5–790.ke 5–790.ne
Total hip arthroplasty	5–820.0
Total hip arthroplasty–special prothesis	5–820.2
Hemiarthroplasty	5–820.4
Total hip arthroplasty–short stem	5–820.9
Other hip arthroplasty	5–820.x

All diagnoses of femoral neck fractures between 2009 and 2019 were included into the study analysis. The cases were divided into male and female subgroups with further classification into age groups.

Categorical data is expressed as frequency counts (percentages). Incidence rates were calculated based on Germany’s historical population aged 20 years or older provided by Destatis. Standardized incidences were calculated for the respective age groups and sex. Data were analyzed using the statistical software SPSS Version 26.0 (IBM, SPSS Inc. Armonk, NY, USA).

## Results

**Fig. 1:**
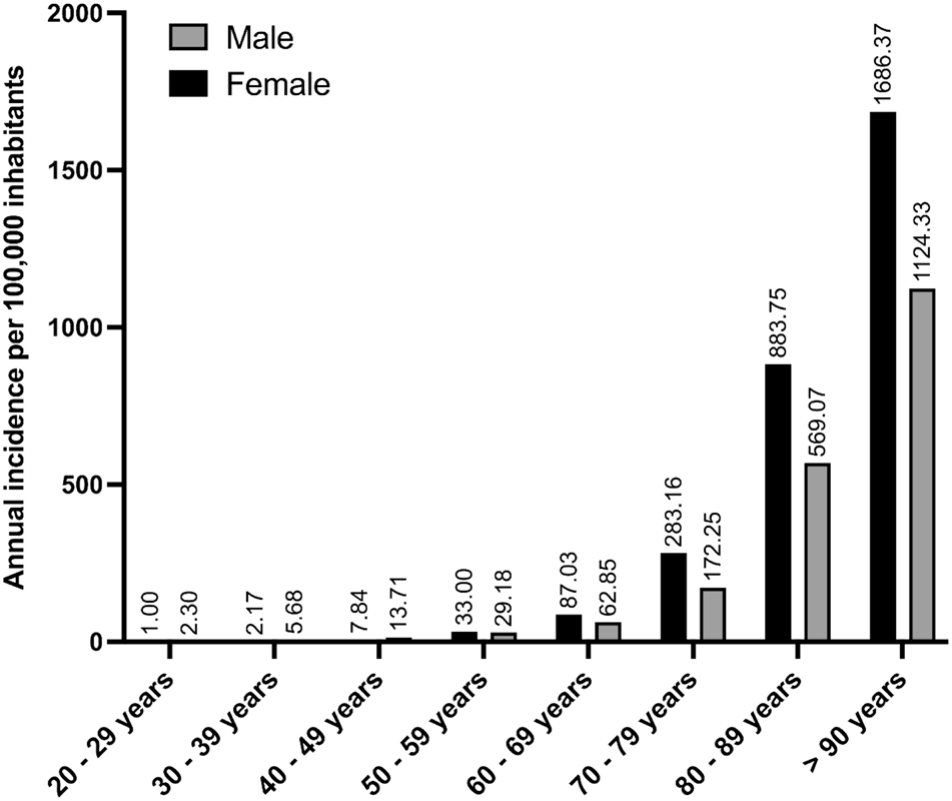
Annual standardized age-adjusted incidences for intracapsular femoral neck fractures in Germany per 100,000 inhabitants as function of sex and age group

Overall, 807,834 intracapsular femoral neck fractures were reported by German medical institutions between 2009 and 2019 and were included in the study analysis. The mean incidence within this eleven-year period was 110.0 fractures per 100,000 inhabitants annually (95% Confidence interval: 109.9–110.1). From a total number of 66,188 and an incidence of 99.7 fractures per 100,000 inhabitants in the year 2009 an increase of 23.2% to 81,570 fractures and an incidence of 120.2 in the year 2019 was documented (Table 2). The proportion of women with intracapsular femoral neck fracture was 68.8% showing an increase of 15.3% over 11 years, while a growth rate of 43.2% in the total amount was registered in the male population. In women, the mean incidence for femoral neck fractures was 147.3 per 100,000 inhabitants, while in men an incidence of 70.6 was documented (Table 3). The incidences grew with increasing patient age, with the highest incidences found in the subpopulation over 90 years of age (women: 1686.4/100,000; men: 1124.3/100,000). In the historical course over eleven years, there was also a continuous increase in all age groups older than 60 years. In patients aging 50 years or older, the incidence of intracapsular femoral neck fracture was higher in women than in men. In patients younger than 50 years the incidence was higher in men than in women (Fig. 1).

**Table 2:** Historic development of population and intracapsular femoral neck fracture prevalence from 2009 through 2019

Year	German population 20 years or older	Total numbers	Incidence per 100,000 inhabitants (95% CI)	Growth rate in % (compared to 2009)
2009	66,400,066	66,188	99.7 (96.2–102.9)	–
2010	66,549,975	69,578	104.6 (99.9–106.7)	5.1
2011	65,398,514	68,671	105.0 (102.1–109.4)	3.8
2012	65,665,069	68,790	104.8 (101.6–109.1)	3.9
2013	65,943,867	71,351	108.2 (103.3–110.9)	7.8
2014	66,677,665	72,727	109.1 (105.6–113.3)	9.9
2015	67,097,676	76,133	113.5 (109.7–117.2)	15.0
2016	67,440,230	75,931	112.6 (109.1–116.6)	14.7
2017	67,540,025	77,590	114.9 (111.1–118.6)	17.2
2018	67,724,921	79,305	117.1 (113.3–120.9)	19.8
2019	67,864,036	81,570	120.2 (116.6–123.9)	23.2

**Table 3:** Historic development from 2009 through 2019 of all intracapsular femoral neck fracture cases divided by age groups. Data is shown as total numbers, percentage in the year in the considered age group

Year	20–29 years Total (percentage)	30–39 years Total (percentage)	40–49 years Total (percentage)	50–59 years Total (percentage)	60–69 years Total (percentage)	70–79 years Total (percentage)	80–89 years Total (percentage)	90 years or older Total (percentage)
2009	173 (0.3)	425 (0.6)	1551 (2.3)	3535 (5.3)	6853 (10.4)	16,577 (25.1)	29,406 (44.4)	7668 (11.6)
2010	178 (0.3)	431 (0.6)	1687 (2.5)	3848 (5.6)	6934 (10.1)	17,927 (26.0)	29,406 (42.6)	8486 (12.3)
2011	161 (0.2)	428 (0.6)	1528 (2.2)	3722 (5.4)	6553 (9.5)	17,608 (25.4)	30,087 (43.4)	9207 (13.3)
2012	171 (0.3)	362 (0.5)	1417 (2.0)	3649 (5.3)	6327 (9.1)	18,159 (26.2)	29,464 (42.5)	9784 (14.1)
2013	142 (0.2)	406 (0.6)	1354 (1.9)	3794 (5.4)	6574 (9.3)	19,075 (26.8)	28,921 (40.9)	10,527 (14
2014	141 (0.2)	405 (0.6)	1253 (1.7)	3802 (5.2)	6475 (8.9)	19,671 (27.1)	29,775 (40.9)	11,205 (15.4)
2015	181 (0.2)	405 (0.5)	1239 (1.6)	4087 (5.4)	6973 (9.1)	20,371 (26.8)	31,270 (41.1)	11,607 (15.3)
2016	162 (0.2)	377 (0.5)	1096 (1.4)	4061 (5.4)	7467 (9.8)	19,504 (25.7)	31,319 (41.3)	11,945 (15.7)
2017	158 (0.2)	375 (0.5)	1115 (1.4)	4122 (5.3)	7795 (10.1)	19,760 (25.5)	32,077 (41.3)	12,188 (15.7)
2018	167 (0.2)	379 (0.5)	1033 (1.3)	4268 (5.4)	8106 (10.2)	19,779 (24.9)	32,810 (41.4)	12,763 (16.1)
2019	168 (0.2)	378 (0.5)	975 (1.2)	4211 (5.2)	8590 (10.4)	19,375 (23.8)	34,924 (42.8)	12,949 (15.9
Growth rate for total number in % (2019 relative to 2009)	– 2.9	– 11.1	– 37.1	19.1	25.4	16.9	18.8	68.9

**Fig. 2:**
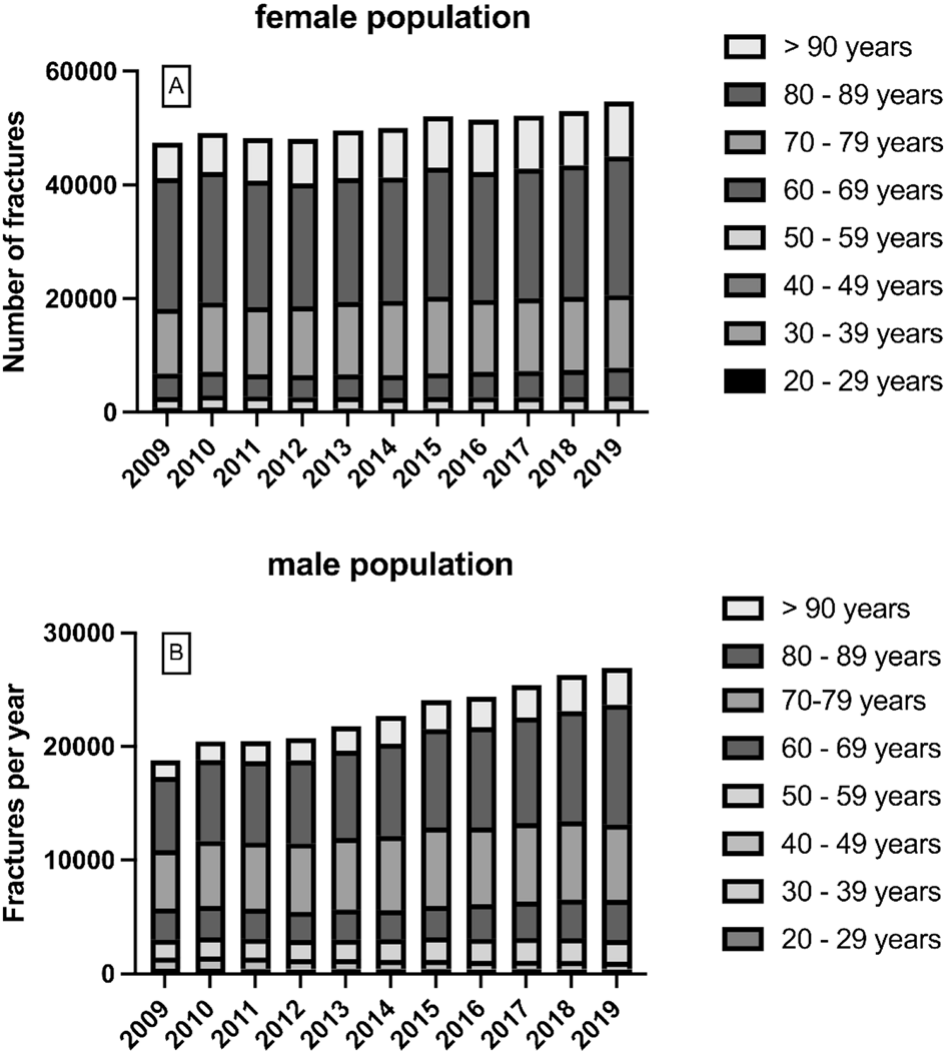
Annual number of intracapsular femoral neck fractures in the female **A** and male **B** population between 2009 and 2019 in Germany

**Fig. 3:**
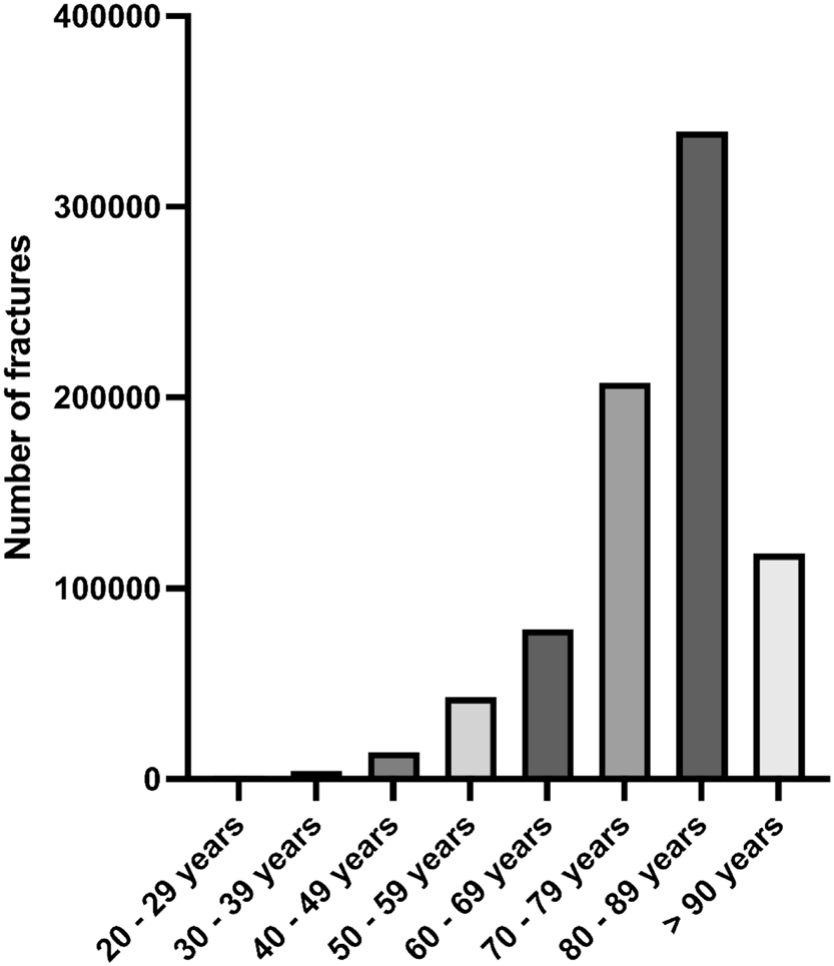
Distribution of intracapsular femoral neck fractures in age classes within the last eleven years in Germany

**Fig. 4:**
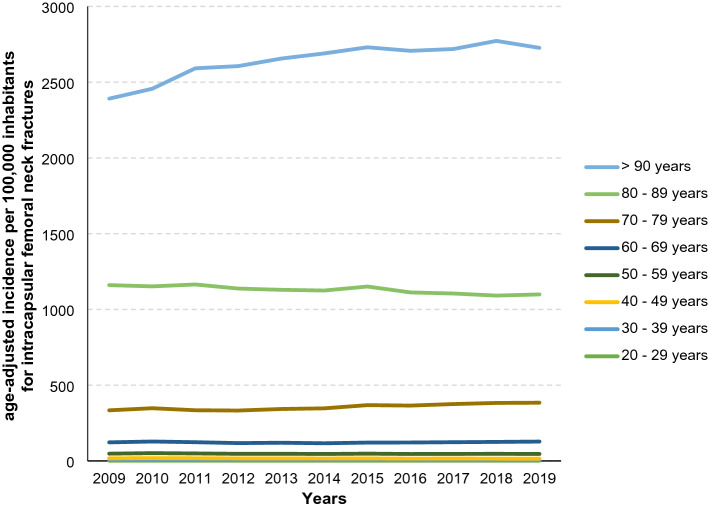
Age-adjusted incidences for intracapsular femoral neck fractures within the time period between 2009 and 2019

Overall, 82.4% (*n* = 665,667) of the patients were 70 years or older. The proportion in woman was thereby at 86.2%, while only 73.9% of the men were 70 years or older (Fig. 2). The majority of the fractures occurred in the age group between 80 and 89 years with 42.0% (*n* = 339,532). For patients aged over 90 years the highest growth of the age-adjusted incidence was found with 32.2%, while in the group between 80 and 89 years a decrease by 13.5% was registered. In the age group between 70 and 79 years a growth of 21.4% and between 60 and 69 years an increase by 9.5% was documented (Table 4; Figs. 3, 4).

**Table 4:** Historic development of age-adjusted incidence per 100,000 inhabitants from 2009 to 2019 for intracapsular femoral neck fracture cases divided by age groups

Year	20–29 years incidence	30–39 years incidence	40–49 years incidence	50–59 years incidence	60–69 years incidence	70–79 years incidence	80–89 years incidence	90 years or older incidence
2009	1.8	4.3	11.2	30.8	74.6	211.4	826.5	1230.9
2010	1.8	4.4	12.3	32.9	76.8	219.8	804.3	1304.4
2011	1.7	4.5	11.6	31.6	74.3	210.9	830.2	1426.9
2012	1.8	3.8	11.1	30.2	70.9	214.9	805.2	1468.4
2013	1.5	4.2	10.9	30.6	72.9	222.8	786.6	1526.7
2014	1.5	4.1	10.5	29.9	70.7	230.5	777.7	1565.2
2015	1.8	4.0	10.8	31.4	73.1	247.3	782.9	1579.3
2016	1.6	3.7	9.9	30.7	75.9	243.7	746.9	1594.8
2017	1.6	3.6	10.4	30.8	77.3	251.8	729.7	1613.9
2018	1.7	3.6	9.9	31.7	78.7	257.3	708.6	1681.2
2019	1.7	3.5	9.6	31.3	81.7	256.6	714.8	1627.5
Growth rate of age-adjusted incidence in % (2019 relative to 2009)	– 5.6	– 18.6	– 14.3	1.6	9.5	21.4	– 13.5	32.2
Incidence rate for male population	2.3	5.7	13.7	29.2	62.9	172.2	569.1	1124.3
Incidence rate for female population	1.0	2.2	7.8	33.0	87.0	283.2	883.7	1686.4

In 70,788 cases in the year 2019 (86.8%) a surgical procedure was documented and analyzed. Thereby, 80.4% of the cases were treated with hip replacement surgery, while osteosynthesis was performed in 19.6% of the treated cases. The most frequently performed surgical procedure in the year 2019 was hemiarthroplasty with 39,001 (56.8%) treated femoral neck fractures. Here, 33,622 (86.2%) hemiarthroplasties included cemented femoral fixation, while 5,389 (13.8%) were classified as uncemented prothesis. Total hip arthroplasty was performed in 15,631 (22.8%) cases. The most frequently performed osteosyntheses for intracapsular femoral neck fractures were fracture reduction and fixation with dynamic compression screw (34.0%, *n* = 4.952), screw (31.3%, *n* = 4.567) and nail (22.2%, *n* = 3.231) (Table 5).

**Table 5:** Performed treatment procedures after intracapsular femoral neck fracture in the year 2019 (*n* = 70,788)

Procedure		Total number (percentage)
Osteosynthesis (*n* = 14,570, 20.6%)	Screw	4567 (6.5)
	Dynamic compression screw	4952 (7.0)
	Wire	267 (0.4)
	Nail	3231 (4.6)
	Fixateur	13 (0.01)
	Plate	1526 (2.2)
	Other osteosynthesis	14 (0.02)
Hip replacement (*n* = 56,218, 79.4%)	Total hip arthroplasty–cemented	3056 (4.3)
	Total hip arthroplasty–partially cemented	4973 (7.0)
	Total hip arthroplasty–uncemented	7611 (10.8)
	Hemiarthroplasty–cemented	33,622 (47.5)
	Hemiarthroplasty–uncemented	5389 (7.6)
	Other prothesis	1567 (2.2)

**Fig. 5:**
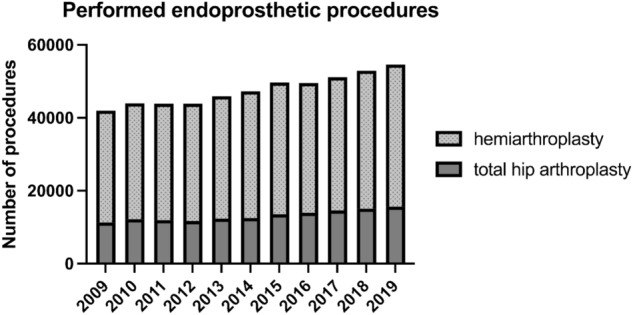
Development of endoprosthetic treatment of intracapsular femoral neck fractures between 2009 and 2019 in Germany

**Fig. 6:**
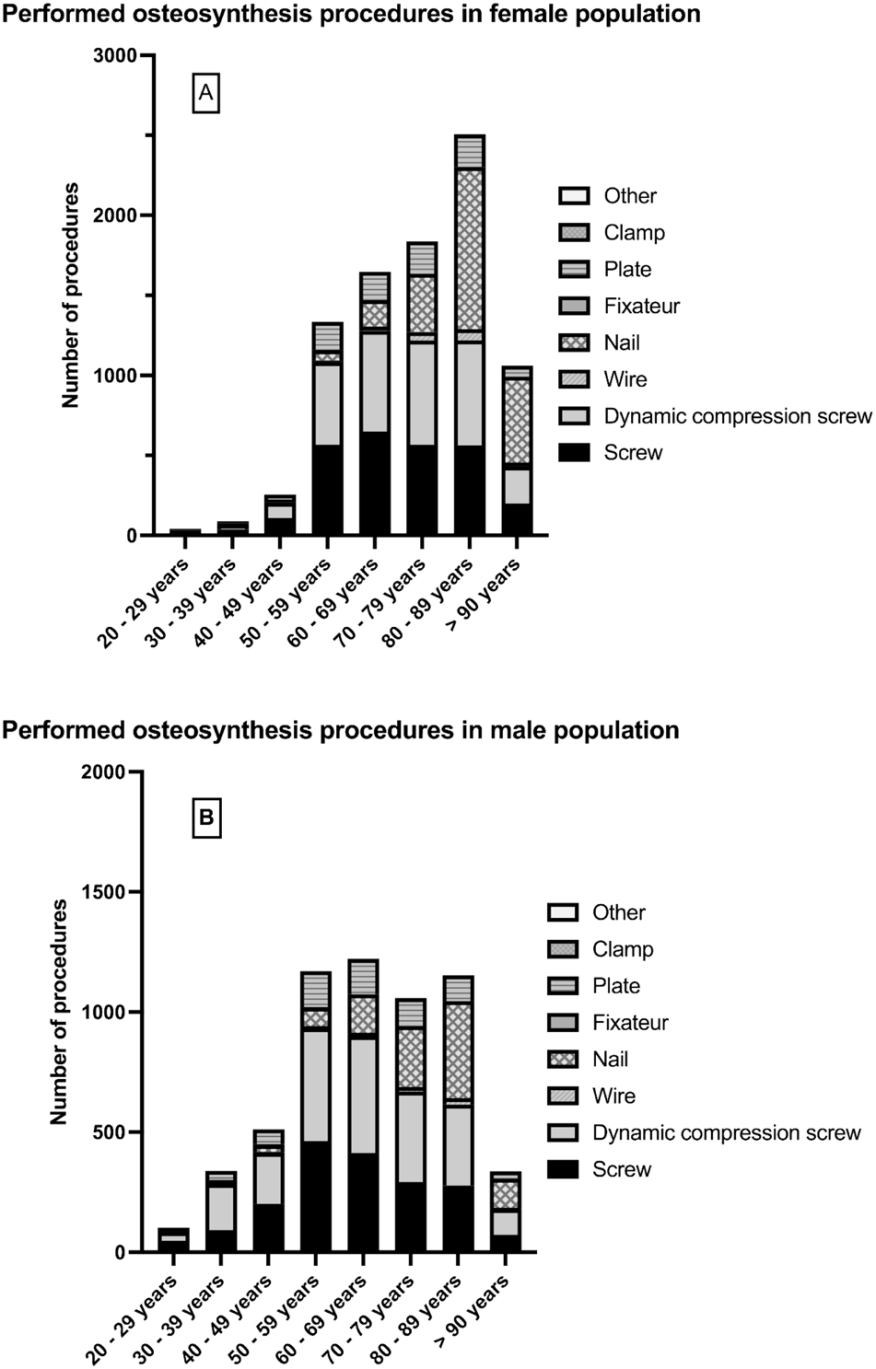
Osteosynthesis procedures after intracapsular femoral neck fractures in women **A** and men **B** of different age classes in Germany

Among the arthroplasty procedures, the highest increase of 107.9% was determined for total hip arthroplasty with a short stem. For conventional total hip arthroplasty, the growth rate was 38.6% and for hemiarthroplasties 27.1% within the period from 2009 to 2019 (Fig. 5). In both women and men, the proportion of fractures treated with nails increased with patient age. While in the age group younger than 70 years the proportion was 12.1% in women and 26.4% in men, it rose to 87.9 and 73.6% respectively in the age group over 70 years. In men, a reduction in the proportion of dynamic compression screws and screw osteosyntheses was also observed with increasing patient age (Fig. 6).

## Discussion

The main finding of our retrospective cohort analysis was the detection of a continuous growth in number and incidence of femoral neck fractures in the female and male population in Germany from 2009 to 2019. With increasing age an increasing incidence was determined, with a clear predominance of female patients. However, in the observation period the number of male patients and incidences in male subgroups–in particular in higher age–were growing fast. With the increasing number of fractures also an increasing number of surgical procedures was detected. Hip replacement surgery was performed more often compared to osteosyntheses. Hemiarthroplasty was the most common performed procedure. Cemented anchorage was the preferred method of femoral shaft fixation.

An increasing number of fractures was already described as a burden for the German and other Western countries´ health care systems. In particular, hip fractures play a major role in the increasingly aging population [1, 2, 12]. For older patients the age-standardized incidence rose particularly with increasing age, mainly in the female population. Female sex, age over 65 years, reduced bone mineral density, chronic diseases, alcohol abuse, tobacco consume, reduced activity level and a decreased body mass index (BMI) were already described as risk factors for the occurrence of femoral neck fractures [4, 10, 16, 17]. Worldwide, the incidence rates of intracapsular femoral neck fractures differ from country to country. Age structure, demographic development and different lifestyle changes are suspected to influence the incidence of intracapsular femoral neck fractures. Recently, a decrease in the likelihood of occurrence was recorded in the United States. A direct correlation with the reduction of tobacco use and a lower rate of alcohol abuse was found as a possible reason for the reduction [10]. Similarly, a reduction in incidence was observed in Finland. In addition to increased strength and functionality in older people, changes in eating habits, prevention programs to prevent falls and osteoporosis (e.g., through bone density measurements, drug therapy), and smoking cessation were listed here as the main factors for the reduction [18]. However, in other societies in Europe and Asia, such as the Netherlands, Italy and Japan, which also have steadily aging populations, incidence rates for intracapsular femoral neck fractures have increased [12–14].

Within the next decades, the population in Germany is expected to decrease, while the age of the population and amount of hip fractures is expected to increase. This issue represents a challenge for the German health care system and was already described for primary joint arthroplasties [19]. Especially in developed countries a similar demographic challenge can be expected. Other factors influencing the incidence rates are migration, which has increased in recent years, especially in Europe, and will have an impact on incidence over the coming years and decades. A prominent finding of our work was the high growth rate of the number of fractures in the male population. With a percentage growth of 43.2%, this was about three times higher than in women in the last decade. The reason for this is the increasing life expectancy in men in Germany, which results in a larger population falling into the risk group. Already in a previous investigation of the German population analyzing the period between 1995 and 2004 a growth by 1% annually has been detected [15]. These findings coincide with our data, where a mean annual growth rate of 2.3% was reported. For patients younger than 50 years a reduction of femoral neck fractures has been already documented for the period between 1995 and 2004 and was confirmed in the last decade [15]. However, due to changes in injury mechanism a comparison to the age group over 50 years is not applicable. High-energy injury mechanisms (e.g. car accidents) are mainly responsible for hip fractures in these age groups. Improved safety precautions can explain the incidence decrease in the younger population [3]. In contrast, older patients mostly suffer from low-energy trauma and usually experience low bone mineral density [3, 16]. Increasing incidence rates with increasing age could be explained by a reduction of bone mineral density caused by lower vitamin D levels and lower BMI, as well as higher risk of falls [5, 20]. To reduce the increasing incidence of intracapsular femoral neck fractures the high-risk group of elderly patients have to be taken care of. Fall prevention programs addressing the mentioned risk factors with systematic implementation of bone density measurement, Vitamin D and calcium supplementation and weaning of psychotropic pharmacologic agents demonstrated additionally to regularly physical exercises a great benefit and potential reduction of fracture incidence [18, 21, 22].

For the treatment of femoral neck fractures, different options of osteosynthesis and prosthetic surgery are available. The poor local blood supply and low amount of cancellous bone are described reasons of a higher malunion risk, avascular osteonecrosis and nonunion after osteosynthesis [3, 16]. Decision making for the appropriate treatment is dependent on patient age and demand, as well as comorbidities and fracture classification. While for osteosynthesis a higher rate of nonunion, necrosis and degenerative changes were described, while arthroplasty implies a higher blood loss and infection rate [3, 7, 16, 23]. In our investigation, a clear trend towards hip replacement surgery is evident. Hemiarthroplasties were the most common used procedure and were accounted for around half of all interventions. Among arthroplasties for the treatment of intracapsular femoral neck fractures also the usage of short stem prothesis showed good results [24]. Even in patients with osteoporotic fractures sufficient results with stable bony integration was found [25]. Among the osteosynthesis the dynamic compression screw was mostly used, but number of procures decreases with increasing patient age. A meta-analysis by Wang et al. [26] demonstrated no significant differences in functional outcome, complication rate and 1-year mortality after the treatment of femoral neck fractures with hemiarthroplasty and total hip arthroplasty. However, a lower re-operation rate was found after total hip arthroplasty, while the rate of dislocation was lower in the group treated by hemiarthroplasty [26]. Within the first years the functional scores and quality of life is similar between both techniques. Intriguingly, after four years a better function was reported for total hip arthroplasties [9]. With regard to these similar functional parameters, but a shorter duration of surgery and thus, a less invasive procedure, the decision to perform a hemiarthroplasty is made more frequently in the trauma situation.

While in the U.S. the majority of hip arthroplasties is performed with cementless fixation of the femoral stem [27, 28], we demonstrated a clear surplus of cemented hemiarthroplasties and total hip arthroplasties in Germany after femoral neck fracture. Almost half of all procedures after femoral neck fracture in our investigation were cemented hemiarthroplasties, while in hemiarthroplasties the proportion of cemented stem fixation was 86.2%. Parker et al. [29] reported a clear advantage of cemented arthroplasties with regard to faster mobilization and reduction of postoperative pain in a systematic review [29]. Furthermore, the rate of aseptic revisions was significantly reduced among cemented hemiarthroplasties and total hip arthroplasties [27, 30–32]. For cemented hemiarthroplasties within the first 2 days after surgery, Fenelon et al. [33] reported an increased mortality compared to uncemented stems. However mortality was not increased seven or 30 days after operative treatment [33]. Data from the NHS demonstrated significantly lower mortality in cemented hemiarthroplasties after 30 days post-surgery and later [34]. While a feared complication of cemented procedures is the increased risk of cardiovascular events, another benefit is the association with a lower number of perioperative fractures [30, 35]. Periprosthetic fractures are the major reason of revision surgery and show a 7% five-year incidence [36]. Another main cause for the revision of an arthroplasty, both hemiarthroplasty and total hip arthroplasty, after fracture treatment is still the occurrence of infection [36]. However, there is a lack of sufficient long-term data on benefits of cemented arthroplasties with regard to infection rates.

Our study has several limitations. A disadvantage of all registry studies is that analysis is based on coding of disease (ICD-10) and procedures (OPS). Only correct coded femoral neck fractures and procedures could be analyzed. Errors in coding, e.g. incorrect classification, could not be evaluated. However, the provided data pool of the Federal Statistical Office of Germany (Destatis) entails comprehensive information about all patients treated for femoral neck fractures in a German hospital in the set time frame. The influence of operation time (day vs. night and weekend vs. weekday) on performed procedure is also a research question, which could not be answered by the study design. Another limitation was that treatment could not be correlated in more detail with patient data, such as comorbidities or ASA score. Therefore, risk and outcome analysis could not be performed. Due to study design, also no individual follow-up of patients with regard to revision or survival rates could be accomplished by our investigation. Fracture registries are able to provide more detailed data and correlation between fracture classification, treatment and comorbidities and should be used for further investigations.

## Conclusion

The total number and the incidence of intracapsular femoral neck fractures has increased within the eleven-year period in Germany between 2009 and 2019. In particular, the male population experienced an increased growth in fracture rate. The most common operative treatment was cemented hemiarthroplasty. An equal trend can be expected for countries with similar demographic characteristics in the future. The introduction and analysis of fracture register data might offer a sufficient database to clarify unanswered questions on evaluation of treatment in the future. In particular in the high-risk group of the elderly population prevention programs to reduce the risk of falling and diminish the risk factors of intracapsular femoral neck fractures should be established.
